# Acellular pertussis vaccines in diverse populations: a review of effectiveness, immunogenicity, and safety

**DOI:** 10.3389/fmicb.2026.1981584

**Published:** 2026-10-30

**Authors:** Yupeng Wang, Shuangxing Li, Kunqi Zou, Boyuan Xuan, Mengchao Zhao, Liyong Yuan, Xingchao Geng

**Affiliations:** 1 Institute for Biological Product Control, National Institutes for Drug Control, NHC Key Laboratory of Research on Quality and Standardization of Biotech Products and NMPA Key Laboratory for Quality Research and Evaluation of Biological Products, Beijing, China; 2 National Center for Safety Evaluation of Drugs, National Institutes for Drug Control, Beijing, China; 3 Department of Pharmacy, General Hospital of Ningxia Medical University, Ningxia, China

**Keywords:** acellular pertussis vaccine, blunting, DTaP, immunogenicity, maternal immunization, safety, Tdap, vaccine effectiveness

## Abstract

Pertussis remains an important vaccine-preventable respiratory disease, particularly in young infants. Because of their lower reactogenicity, acellular pertussis vaccines have replaced whole-cell vaccines in many countries and are widely used for infant primary immunization, booster vaccination in children, adolescents and adults, and maternal immunization. However, pertussis resurgence, waning immunity, antigen-deficient circulating strains and limited effects on nasopharyngeal colonization have renewed interest in antigen composition, immune mechanisms and clinical protection. This narrative review synthesizes evidence on acellular pertussis vaccines across different populations, focusing on: clinical protection and waning after infant and childhood vaccination; immunogenicity and safety of adolescent and adult boosters; maternal vaccination and its effects on early infant protection and subsequent immune responses; and product-specific evidence on combination vaccination and coadministration. Rather than attempting an exhaustive comparison of all products and studies, this review highlights representative clinical evidence and distinguish clinical outcomes from immunogenicity and safety endpoints. Current data indicate that pertussis toxin-containing acellular vaccines induce clear antibody responses, and multicomponent formulations broaden antigenic coverage. Three-component or higher acellular vaccines have shown efficacy of approximately 84–85% against typical childhood pertussis, but protection after DTaP wanes over time. DTaP and Tdap are generally well tolerated; most adverse events are mild-to-moderate local or transient systemic reactions. Maternal Tdap improves early infant antibodies but may blunt some subsequent infant responses. Future research should define correlates of protection, evaluate antigen variation, and strengthen evidence on mucosal immunity, coadministration, real-world effectiveness and long-term safety.

## Graphical abstract

**Figure:**
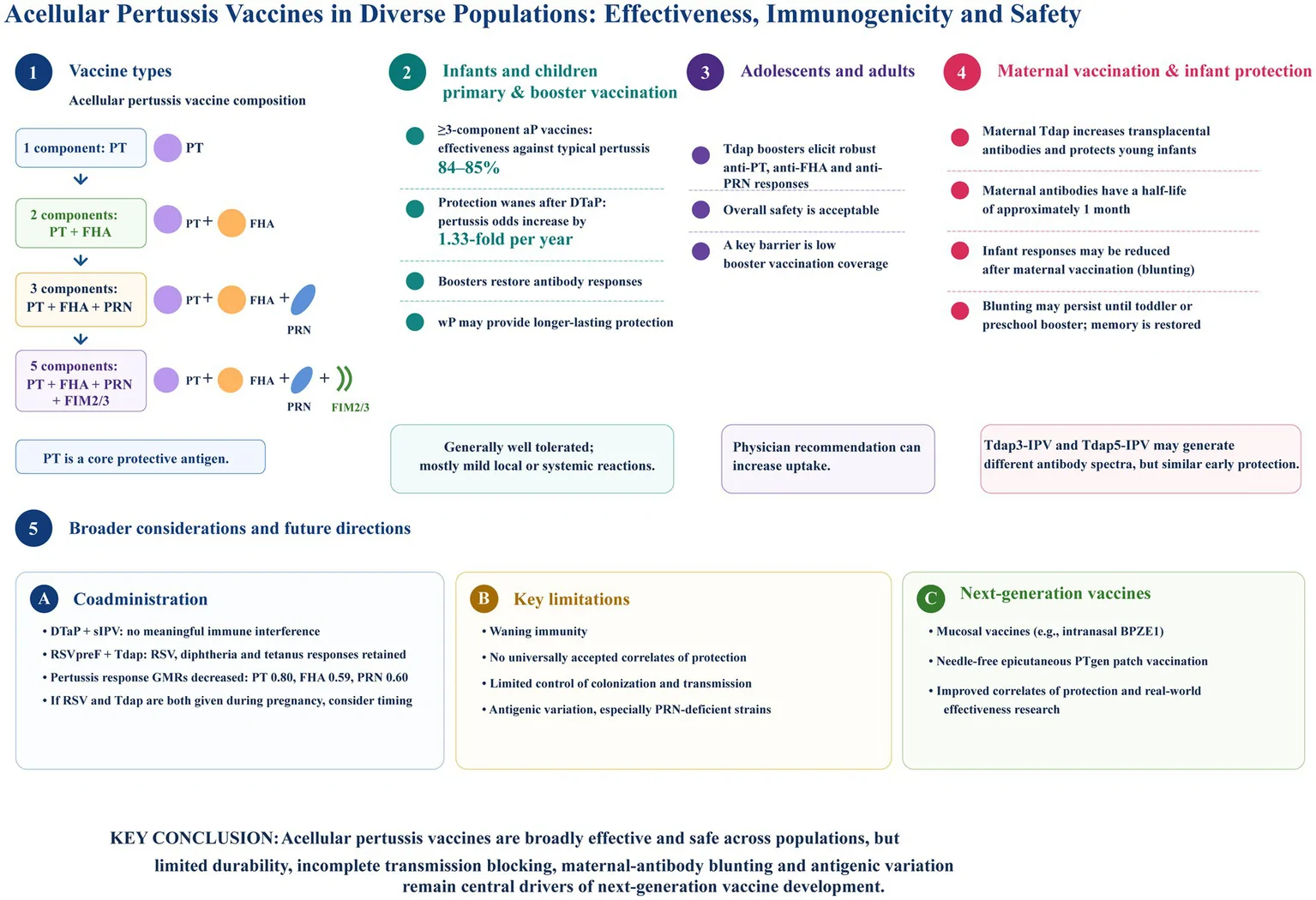

## 1 Introduction

Pertussis, caused by *Bordetella pertussis*, is a highly contagious acute respiratory disease. Although pertussis-containing vaccines have been incorporated into childhood immunization programs in most countries, the disease continues to occur in infants, adolescents and adults, and young infants remain at highest risk of severe disease, hospitalization and death. Vaccination is therefore the cornerstone of pertussis prevention. Whole-cell pertussis vaccines historically provided substantial clinical protection, but their relatively high reactogenicity led many countries to adopt less reactogenic acellular pertussis (aP) vaccines (Aronsson et al., 2022; Yu et al., 2025; Duda-Madej et al., 2025). The WHO 2015 position paper describes the different aP vaccine formulations and pertussis immunization strategies, Vaccine products and national schedules vary. Pertussis vaccination coverage also varies across countries and populations. WHO/UNICEF estimated that, in 2024, global coverage was 89% for the first dose and 85% for the third dose of a diphtheria-tetanus-pertussis-containing vaccine. Reported pertussis burden is further affected by access to health services, surveillance, and laboratory diagnosis. PCR-based diagnosis has become an important tool, but diagnostic capacity varies across and within countries. A systematic review of laboratory-confirmed pertussis in low and middle-income countries found that available studies were concentrated in upper-middle-income settings and identified improved access to PCR-based diagnosis as a surveillance need (Muloiwa et al., 2020).

An additional challenge in interpreting pertussis vaccine evidence is the absence of an unequivocally established immunological correlate of protection. Antibody concentrations against pertussis toxin (PT), filamentous hemagglutinin (FHA), pertactin (PRN), and fimbrial antigens are used to characterize vaccine-induced responses, but no universally accepted antibody threshold can be used to infer clinical protection. This limits the interpretation of immunogenicity findings and immune-bridging studies. Such studies can support immunological comparisons within an appropriate product and comparator framework, but do not by themselves establish equivalent clinical protection across different vaccine formulations or platforms.

Despite these limitations, epidemiological and seroepidemiological data provide important information on pertussis circulation and population immunity. In China, a systematic review of anti PT IgG seroprevalence including 39 studies and 47,778 participants, found a pooled seropositivity of 7.06% (95% CI: 5.50-9.07%), while reported incidence increased from 0.12 per 100,000 in 2013 to 2.69 per 100,000 in 2022(Zhu et al., 2024). Global anti-PT IgG serosurveillance from 2010 to 2023 also showed regional variation and substantial heterogeneity (Ma et al., 2026). These findings should be interpreted in the context of differences in vaccination schedules, coverage, case ascertainment, and diagnostic capacity.

Acellular pertussis vaccines are based on selected protective antigens of *B. pertussis*, including PT, FHA, PRN, and fimbrial antigens FIM2 and FIM3. According to antigen composition, aP vaccines can be classified as one-, two-, three or five-component products. However, product comparisons should not be based on component number alone, because antigen dose, PT detoxification method, manufacturing process, adjuvant, combination antigens, and immunization schedule also differ (Olin et al., 1997). The clinical efficacy trials of aP vaccines were conducted mainly in the 1990s, and their findings were specific to the products, study designs, and case definitions evaluated; they should not be interpreted as direct comparisons of all currently available products (Zhang et al., 2011).

Recent pertussis-vaccine research has not focused exclusively on reducing reactogenicity. It also addresses the quality and durability of protection, real-world vaccine effectiveness, antigenic variation, and immune responses beyond serum antibody concentrations. Real-world studies complement randomized trials by assessing vaccine performance under specific routine conditions (Wilkinson et al., 2021). Cellular immune responses are also under investigation, although their relationship to durable clinical protection remains to be established. Alternative approaches, including the live attenuated nasal vaccine BPZE1 and epicutaneous PTgen delivery, are being studied to explore immune responses beyond those elicited by conventional intramuscular aP vaccines (Chatzis et al., 2021; Gbesemete et al., 2025).

Given these issues, this narrative review synthesizes representative evidence on aP vaccines according to antigen composition, target population, immunization schedule and main outcomes. We searched PubMed for literature published from January 2021 through June 2026, using combinations of MeSH terms and other terms related to pertussis vaccines, including DTaP and other aP formulations. In PubMed the search strategy was: (((((Pertussis Vaccine[MeSH Terms]) OR (Diphtheria-Tetanus-acellular Pertussis Vaccines[MeSH Terms])) OR (Vaccine, Pertussis[Other Term])) OR (DTaP Vaccine[Other Term])) OR (Diphtheria Tetanus acellular Pertussis Vaccines[Other Term])) OR [ACEL-IMUNE(Other Term)]. We selected clinically relevant studies that provided information on vaccine composition, target population, immunization schedule, effectiveness, immunogenicity, or safety. Earlier landmark studies were also cited where needed to describe the historical development of aP vaccines and the evidence from pivotal efficacy trials. This review focus on infants, children, adolescents, adults and pregnant women, and discusses the limitations of current evidence and priorities for future research.

## 2 Antigen composition and types of acellular pertussis vaccines

The defining feature of acellular pertussis vaccines is the use of selected protective antigens rather than whole inactivated bacteria. PT is the central antigen in currently licensed and investigational acellular vaccines. As an important *B. pertussis* virulence factor, PT mediates multiple systemic pathological effects, and anti-PT antibodies are widely used as a core indicator of vaccine-induced pertussis immunity. Additional adhesion-related antigens, including FHA, PRN and FIM2/FIM3, broaden coverage against bacterial adhesion, colonization and fimbrial immune responses (Goldsmith et al., 2025; Gorringe and Vaughan, 2014).

By antigen composition, acellular pertussis vaccines are commonly grouped as one-component (PT), two-component (PT + FHA), three-component (PT + FHA + PRN) and five-component (PT + FHA + PRN + FIM2 + FIM3) vaccines. One-component vaccines induce anti-PT responses only and therefore provide the narrowest antigenic coverage. Two-component vaccines additionally induce anti-FHA responses. Three-component vaccines include PRN and are frequently evaluated in DTaP and Tdap studies, including maternal immunization studies. Five-component vaccines further include FIM2 and FIM3 and may expand coverage of fimbrial antigens.

In practice, acellular pertussis vaccines are frequently used in combination products. Infants and children receive DTaP or DTaP-containing pentavalent or hexavalent vaccines. Adolescents and adults generally receive reduced-antigen-content Tdap boosters. Pregnant women receive Tdap to transfer maternal antibodies across the placenta and protect infants before completion of primary immunization. BPZE1 is not a traditional purified-component acellular vaccine, but its ability to induce mucosal immunity and reduce nasopharyngeal colonization makes it an important next-generation candidate (Gbesemete et al., 2025; Jones et al., 2021; Klein et al., 2019). Epicutaneous PTgen patch vaccination also suggests that future pertussis boosters may not be restricted to intramuscular injection (Chatzis et al., 2021). For BPZE1, mucosal IgA and nasal colonization or bacterial-load measures provide information on mucosal responses and infection-related outcomes, but they have not yet been established as reliable predictors of reduced onward transmission. For the PTgen epicutaneous patch, early studies primarily assess safety and immune responses; the ability of these responses to predict protection against infection, transmission, or clinical disease remains undetermined.Therefore, comparisons across aP vaccines should consider not only component composition but also antigen dose, PT detoxification method, adjuvant, combination antigens, immunization schedule and target population. Table 1 presents selected randomized and controlled clinical studies published from 2021 to 2026 to highlight recent research across populations and vaccination settings. This period was selected to summarize recent clinical developments; it applies to the studies presented in Table 1 and does not define the date range of evidence discussed throughout the review. Earlier pivotal efficacy trials and other landmark studies are discussed in the relevant sections of the main text. Table 1 is intended to illustrate the range and heterogeneity of recent studies, rather than to provide an exhaustive catalogue or a direct comparison of vaccine products.

**Table 1:** Selected randomized and controlled studies of different acellular pertussis vaccines in different populations (2021–2026).

Population	Vaccine composition	Schedule	Main outcomes	Clinical implication	Publication year/Country
Children/adolescents	Co-purified DTaP	Single booster dose	Induced anti-PT and anti-FHA antibody responses without impairing DT and TT responses.	Supports the feasibility of replacing DT with co-purified DTaP as a booster strategy in 6-year-old children in China.	2026, China (Tang et al., 2026)
Children/adolescents	Monovalent three-component aP + HBV; DTaP-HBV-Hib-IPV; DTPa/dTpa	aP + HBV at birth; DTaP-HBV-Hib-IPV at 2, 4 and 6 months; DTPa/dTpa at 18 months or 4 years	Marked anti-PT, anti-FHA and anti-PRN responses; booster responses remained good at 18 months and 4 years.	Neonatal aP vaccination may be a potential strategy when maternal Tdap is absent or maternal antibodies are insufficient.	2025, Australia (McAlister et al., 2025)
Children/adolescents	Five-component Tdcp	Single booster dose	Strong antibody responses, particularly to pertussis antigens.	Provides preliminary evidence for pertussis booster strategies in people aged > = 6 years in China.	2025, China (Liu et al., 2025)
Children/adolescents	DTaP, sIPV and MMR	Concomitant versus separate administration	Concomitant administration did not meaningfully impair immunogenicity; DTaP-related responses met non-inferiority criteria.	Supports concomitant sIPV, DTaP and MMR vaccination at 18 months in China.	2023, China (Xu et al., 2023)
Adolescents/adults	BPZE1	Intranasal vaccination followed by controlled human infection challenge with *B. pertussis* B1917	Induced nasal IgA, serum IgA/IgG and bactericidal antibodies and reduced nasal bacterial load.	A candidate mucosal vaccine that may overcome limited effects of conventional aP vaccines on infection and transmission.	2025, UK (Gbesemete et al., 2025)
Adolescents/adults	Tdap-1018	Single 0.5 mL intramuscular dose	Induced stronger anti-PT responses than Boostrix.	Supports further development of the 3,000 ug Tdap-1018 formulation as a next-generation booster candidate.	2024, Australia (Richmond et al., 2024)
Adolescents/adults	Tdapgen, apgen and Tdapchem	Single intramuscular dose	Tdapgen and apgen met non-inferiority criteria and showed trends toward superiority; responses and persistence were better than Tdapchem.	Genetically detoxified recombinant low-dose pertussis vaccines showed favorable immunogenicity and safety in Thai adolescents aged 9–17 years.	2024, Thailand (Puthanakit et al., 2024)
Adolescents/adults (4–65 years old)	SIIPL Tdap	Single 0.5 mL intramuscular dose	Diphtheria and tetanus seroprotection and pertussis booster responses met non-inferiority criteria.	SIIPL Tdap was non-inferior to Boostrix in healthy people aged 4–65 years.	2023, Germany (Aydin et al., 2023)
Adolescents/adults	PTgen	Needle-free epicutaneous delivery	Viaskin-PT was safe in healthy adults; effective immune activation depended on laser skin microporation.	Provides a needle-free, non-traditional-adjuvant approach for reactivating anti-PT immune memory.	2020, Switzerland (Chatzis et al., 2021)
Mother-infant pairs	Tdap-IPV; DTwP-HBV-Hib; DTaP-HBV-Hib-IPV	Maternal Tdap-IPV at 28–34 weeks; infant primary vaccination at 8, 12 and 16 weeks with wP or aP-containing vaccines	Maternal Tdap-IPV increased pre-primary infant serum anti-PT IgG, PT neutralization, whole-cell IgG binding, SBA and nasal pertussis-specific IgG; DTwP induced stronger responses than DTaP.	Supports early systemic and mucosal immune responses following maternal and infant vaccination; clinical protection was not assessed in this immunological substudy.	2026, The Gambia (Saso et al., 2026)
Mother-infant pairs	TdaP containing genetically detoxified PT	Td at 16–26 weeks and TdaP at 20–29 weeks in pregnant women with or without HIV	Induced anti-PT and anti-FHA responses in pregnant women.	Provides direct evidence for genetically detoxified TdaP in areas with high HIV prevalence.	2025, Uganda (Nakabembe et al., 2025)
Mother-infant pairs	Tdap; DTwP-HB-Hib; DTaP-HB-Hib-IPV	Maternal Tdap at 27–36 weeks; infant doses at 2, 4 and 6 months plus 18-month booster	Higher maternal antibody levels were associated with lower infant fold-rises after subsequent vaccination.	Maternal antibodies may affect both aP and wP infant responses, particularly to PT, DT, PRN and FHA.	2025, Thailand (Sereeyothin et al., 2025)
Mother-infant pairs	Tdap5; DTaP3; DTaP5	Maternal Tdap5 at 30 weeks; infant DTaP3 or DTaP5 at 2, 4 and 6 months plus booster at 12 months	Infants in the maternal Tdap5 group had higher antibodies at birth and 2 months; blunting varied by maternal and infant formulations.	Blunting is influenced by the combination of maternal and infant vaccine products.	2024, Canada(Brousseau et al., 2024)
Infant (2-<43 Months)	DPT-IPV-Hib	Primary series every 3–8 weeks in infants aged ≥2 and <7 months; booster 6–13 months after primary series	Immunogenicity met non-inferiority criteria after primary vaccination; all tested antibodies reached 100% seropositivity after booster.	The Japanese pentavalent Gobik vaccine showed good immunogenicity and acceptable safety.	2023, Japan (Nakano et al., 2024)
Infant	DTwP-Hib-HepB; DTaP-Hib-HepB-IPV	wP/aP/aP mixed schedule beginning at 6 weeks versus standard aP/aP/aP schedule; booster at 18 months	Mixed wP/aP schedule met non-inferiority for anti-PT IgG but had higher reactogenicity.	The mixed schedule was immunogenic for PT but requires reactogenicity consideration.	2024 Australia (Pérez Chacón et al., 2024)
Mother-infant pairs	DTaP-IPV; DTaP-Hib-HepB-IPV	Maternal vaccination at <=23 + 6, 24–27 + 6 or 28–31 + 6 weeks; infant vaccination at 2, 3 and 4 months	Infant anti-PT and anti-PRN levels, seropositivity and placental transfer were similar across gestational timing groups; infant post-primary levels did not differ substantially.	Supports a flexible 16–32 week maternal vaccination window.	2023, UK (Calvert et al., 2023)
Mother-infant pairs	dTaP3-IPV; dTaP5-IPV	Maternal vaccination with three or five-component pertussis vaccine; preschool booster with dTaP5-IPV	Anti-PT blunting may persist until preschool age but was largely overcome after booster vaccination.	Maternal immunization can influence preschool pre-booster antibody levels, but booster vaccination restores responses.	2021, 2022, UK (Jones et al., 2021; Sapuan et al., 2022)
Infant	DTcP	Infants aged 2 months vaccinated on 2–3-4 or 2–4-6 month schedules	Seroconversion met non-inferiority criteria; seropositivity exceeded 96%; GMCs were higher with the 2–4-6 month DTcP schedule.	A three-component genetically engineered DTcP vaccine in Chinese infants was safe and immunogenic.	2026, ChinaZhang et al. (2026)
Infant	DTaP plus sIPV	Concomitant or separate DTaP and sIPV in infants aged 60–90 days	Immunogenicity and seroconversion after concomitant administration were non-inferior to separate administration.	Supports same-day sIPV and DTaP administration in Chinese infants.	2022, ChinaSun et al. (2022)

aP, acellular pertussis; DTaP, diphtheria, tetanus and acellular pertussis vaccine; DTwP, diphtheria, tetanus and whole-cell pertussis vaccine; FHA, filamentous hemagglutinin; FIM, fimbriae; GMC, geometric mean concentration; GMR, geometric mean ratio; PRN, pertactin; PT, pertussis toxin; Tdap, tetanus, reduced diphtheria and acellular pertussis vaccine; TT, tetanus toxoid; wP, whole-cell pertussis. The studies summarized in Table 1 differ in immunization schedules, numbers and intervals of doses, sampling time points, laboratory assays, response thresholds, and outcome measures. Their immunogenicity results should therefore not be compared directly across studies or interpreted as equivalent evidence of clinical protection. Cellular or mucosal immune responses and controlled human challenge outcomes are distinct endpoints and should be interpreted according to their respective study designs. For Tdap-1018, 3,000 μg refers to the dose of the CpG 1,018 adjuvant.

## 3 Effectiveness and immunogenicity in infant primary and childhood booster immunization

Infant and childhood studies provide important evidence on the clinical protection afforded by pertussis vaccines. The pivotal randomized efficacy trials of aP vaccines were conducted mainly in the 1990s and evaluated specific products using study-specific populations and case definitions. Long-term follow-up reports and subsequent studies add evidence on protection after primary vaccination, but results from different trials should not be interpreted as direct comparisons of all aP products or as evidence that antigen component number alone determines effectiveness (Aronsson et al., 2022; Salmaso et al., 2001).

A Cochrane review of six efficacy trials involving 46,283 participants reported efficacy of 84-85% for aP vaccines with three or more components against typical childhood pertussis and 71–78% against mild pertussis. For one or two-component vaccines, reported efficacy was 59–75% against typical disease and 13–54% against mild disease (Zhang et al., 2011). Historical efficacy estimates depend on the clinical case definition used. The WHO case definition for typical pertussis generally required at least 21 days of paroxysmal cough together with laboratory confirmation or an epidemiological link to a confirmed case. Less stringent definitions of mild or less-severe pertussis included broader cough criteria, which varied across trials. The Cochrane review reported ranges of efficacy estimates from historical trials, but differences in trial design and case definitions limit direct comparisons across studies. These estimates reflect the products, case definitions, and trial conditions included in the review and should not be generalized to every vaccine within a component category.

Household-exposure studies provide additional evidence under conditions of intense exposure. Older studies reported protection following a monocomponent PT toxoid vaccine, with estimates varying by dose number, exposure setting, and case definition (Heininger et al., 1998; Storsaeter et al., 1998). These studies remain informative as historical evidence but do not provide direct comparisons of current vaccine products.

Protection after both wP and aP vaccination can decline over time. Observational evidence and systematic reviews indicate that pertussis vaccine effectiveness wanes after primary vaccination, with faster waning generally reported after aP schedules than after wP schedules, although estimates vary considerably across studies and settings (Wilkinson et al., 2021). The long-term Swedish follow-up and other historical studies should therefore be interpreted alongside more recent epidemiological evidence (Aronsson et al., 2022). Estimates of waning based on models or assumptions should be identified as such; for example, the estimate that approximately 10% of children might remain protected 8.5 years after vaccination is model-based and depends on assumptions about initial vaccine effectiveness (McGirr and Fisman, 2015) (Gao et al., 2022).

Childhood booster studies show that booster vaccination restores antibody responses to PT, FHA, PRN and FIM. However, local reactogenicity, particularly injection-site pain, erythema and swelling, is more common after booster doses than after infant primary vaccination (Tang et al., 2026; Liu et al., 2025). Evaluation of pediatric acellular pertussis vaccines should therefore integrate short-term effectiveness, antibody persistence, booster responsiveness and reactogenicity.

## 4 Booster immunization in adolescents and adults

Because pertussis continues to circulate among adolescents and adults, Tdap or dTpa booster immunization is an important part of pertussis-vaccine research and public-health strategy. Adolescents and adults may experience prolonged cough illness and can also serve as sources of infection for young infants. Booster immunization therefore has both individual and potential population-level value.

Randomized and non-inferiority studies consistently show that Tdap/dTpa boosters increase antibodies to PT, FHA, PRN and FIM. Comparisons across products generally use immunobridging or non-inferiority designs, with endpoints such as geometric mean concentration (GMC), seroresponse rate, fold-rise after booster vaccination and non-inferiority to licensed comparator vaccines. Most Tdap studies meet predefined immunogenicity criteria, supporting their feasibility as boosters in adolescents and adults (Richmond et al., 2024; Puthanakit et al., 2024; Aydin et al., 2023; Sricharoenchai et al., 2018).

Adult studies also include healthcare workers, older adults and people with special exposure risks. Vaccination of healthcare workers can increase pertussis-specific humoral immunity and may theoretically reduce nosocomial transmission. Studies in older adults show that Tdap is generally immunogenic and acceptable in terms of safety, although immunosenescence, comorbidities and prior vaccination history may influence antibody levels and persistence (Grimprel et al., 2005; Hara et al., 2013; Van Damme et al., 2011; Weston et al., 2012).

Coverage remains a key challenge. In a French intervention study targeting 25-year-old adults, overall dTcaP vaccine claim within 12 months was only 16.7%; uptake was 17.4% in the intervention group and 15.5% in the control group, with no significant difference (*P* = 0.29). General-practitioner consultation within 12 months also did not differ significantly, but participants who consulted a general practitioner were more likely to claim dTcaP vaccine. These findings suggest that face-to-face physician recommendation and prescription may be more effective than letters, e-mails or text-message reminders alone (Hurtaud et al., 2023).

Overall, evidence supports the immunogenicity and safety of Tdap boosters in adolescents and adults, but improving coverage, extending durability and reducing transmission to vulnerable infants remain important research and implementation priorities.

## 5 Maternal immunization and early infant protection

The purpose of maternal Tdap/dTpa immunization is to transfer maternal antibodies across the placenta and protect infants before completion of their own primary series. Randomized and follow-up studies show that maternal Tdap increases maternal, cord-blood and early infant antibodies against PT, FHA, PRN or FIM (Sereeyothin et al., 2025; Brousseau et al., 2024). In several studies, anti-PT GMCs at birth were substantially higher in infants born to vaccinated mothers than in controls, and placental transfer was efficient(Jones et al., 2021; Daniel et al., 2025).

The passive protection achieved after maternal vaccination has a defined time window. An individual participant data meta-analysis of 1,426 mother-infant pairs found that maternally derived antibodies had short half-lives, ranging from 28.7 days (95% CI: 24.4-35.0) for tetanus antibodies to 35.1 days (95% CI: 30.7-41.1) for PRN antibodies; PT, FHA and FIM2/3 antibodies also decayed on the scale of approximately one month (Oguti et al., 2022). Thus, maternal Tdap primarily protects infants in the earliest postnatal period, especially before completion of primary immunization.

Timing of vaccination can affect antibody generation and transfer. If vaccination occurs too close to delivery, there may be insufficient time for maternal antibody production and transplacental transfer (Jones et al., 2021; Daniel et al., 2025; Barug et al., 2019). The Optimum randomized equivalence trial provides evidence on the immunogenicity of maternal pertussis vaccination at different gestational ages, but it does not establish a universally optimal vaccination window. In this trial, vaccination between 16 and 32 weeks resulted in equivalent cord-blood anti-pertussis toxin and anti-pertactin IgG concentrations across the studied groups; however, anti-filamentous haemagglutinin IgG concentrations were higher after vaccination at 28–32 weeks than after vaccination before 24 weeks. No differences in infant antibody GMCs after completion of the primary immunization series were observed between the maternal vaccination-timing groups. These findings are based primarily on immunological outcomes and should not be interpreted as demonstrating equivalent clinical protection at every gestational age (Calvert et al., 2023). Vaccination earlier in pregnancy may provide more opportunity for maternal antibody production and placental transfer before a possible preterm delivery, and may reduce the chance that vaccination is missed if delivery occurs before the later recommended window. Conversely, an earlier dose allows more time for maternal antibody concentrations to decline before birth. The limited number of preterm infants in OpTIMUM prevents firm conclusions about the optimal timing for this group. The trial also found no difference in infant post-primary antibody GMCs across the vaccination-timing groups, but this does not establish that timing has no effect on the clinical consequences of blunting.

Recommendations should therefore be interpreted in the context of each country’s immunization program and supporting evidence. The OpTIMUM findings support consideration of a broader window under the conditions studied, but do not by themselves justify replacing national recommendations. Clinical guidance should balance timely vaccine delivery, the opportunity to protect infants born preterm, antibody transfer and persistence, and the limitations of available clinical-outcome data.

Maternal Tdap may modulate subsequent active immune responses to infant vaccination. In this context, blunting refers to lower antibody geometric mean concentrations or responses to one or more pertussis antigens after infant vaccination among infants whose mothers received Tdap during pregnancy, compared with infants whose mothers did not. Infants of vaccinated mothers have higher pertussis-specific antibody concentrations at birth and during early infancy, but some studies have reported lower antibody concentrations after subsequent DTaP-, DTwP-, or pertussis-containing combination vaccines (Jones et al., 2021; Sapuan et al., 2022; Abu-Raya et al., 2021). Because no universally accepted correlate or threshold of protection against pertussis has been established, these lower antibody measurements do not by themselves demonstrate reduced clinical protection. Follow-up studies have reported that differences in selected antibody responses may persist until a later booster, although infants in both groups generally mount strong responses after booster vaccination (Martinón-Torres et al., 2021).

The clinical significance of blunting remains uncertain and should be considered alongside the risk faced by young infants before they are eligible to receive their first pertussis vaccine dose. These infants are at greatest risk of severe disease and pertussis-related death. Maternal Tdap promotes transplacental transfer of antibodies and provides early protection during this vulnerable period; observational evaluations have also reported reductions in pertussis cases and hospitalizations among young infants following implementation of maternal vaccination programs (Amirthalingam et al., 2014). Current evidence does not establish that blunting results in increased breakthrough pertussis, hospitalization, or severe disease after infant vaccination. Thus, the demonstrated value of maternal vaccination in protecting infants early in life should be weighed against a possible later effect whose clinical importance has not been established. Continued follow-up of infant disease outcomes is needed to clarify this balance.

## 6 Immune interference in combination vaccines and co administration

Acellular pertussis antigens are commonly delivered as DTaP, Tdap or multivalent combination vaccines. Pediatric combination vaccines, such as DTaP-IPV/Hib and DTaP-HBV-IPV/Hib, can reduce the number of injections while inducing responses to multiple antigens. Most major antigen responses meet predefined immunological criteria. However, immune interference can occur and depends on antigen content, adjuvant, formulation and schedule. Interpretation should therefore be product-specific (McAlister et al., 2025; Xu et al., 2023).

A Chinese study of concomitant Sabin-strain inactivated poliovirus vaccine (sIPV) and DTaP in infants found no significant differences in poliovirus, diphtheria, tetanus or pertussis antibody responses between concomitant and separate administration, and non-inferiority criteria were met. Local and systemic reactions were slightly more frequent with concomitant administration but were mostly mild or moderate, and no substantial immune interference was observed (Xu et al., 2023).

Adult coadministration studies have evaluated Tdap with poliovirus, measles-mumps-rubella and other vaccines. Most studies suggest that coadministration is feasible and that major antigen responses can meet predefined criteria, although local or systemic reactogenicity may be additive. However, not all coadministration preserves pertussis-antigen responses. In a phase IIb study of RSVpreF coadministered with Tdap in non-pregnant women, RSV neutralizing antibodies and diphtheria and tetanus responses were not impaired, but the GMRs for anti-PT, anti-FHA and anti-PRN were 0.80, 0.59 and 0.60, respectively, and did not meet non-inferiority criteria (Peterson et al., 2022). Accordingly, immunogenicity and safety should be evaluated for each specific vaccine combination and relevant target population. Clinical recommendations should take account of direct evidence for the combination under consideration, including the population, formulation, administration schedule, measured endpoints, and applicable national guidance. Findings from one coadministration study should not be assumed to apply to other vaccine combinations.

Thus, the key question for combination vaccines and co administration is not simply whether vaccines can be given at the same visit, but whether improved convenience and coverage are achieved while maintaining adequate responses to critical antigens and acceptable safety. Future schedules should be evaluated for specific vaccine combinations and target populations rather than extrapolated from unrelated combinations.

## 7 Safety profile

A major reason for replacing whole-cell pertussis vaccines with acellular products was reduced reactogenicity. Studies in children, adolescents, adults and pregnant women generally show that acellular pertussis vaccines have acceptable safety profiles. Common adverse events include injection-site pain, erythema and swelling, as well as transient systemic symptoms such as fever, fatigue, irritability, headache or myalgia. Most events are mild to moderate and resolve within a short period (Jones et al., 2021; Puthanakit et al., 2024; Aydin et al., 2023; Zhang et al., 2026).

In children and infants, local reactions remain the principal safety burden after combination or booster vaccination. In a Chinese study of co-purified DTaP booster vaccination at six years of age, vaccine-related adverse events within 28 days occurred in 23.75% (57/240) of the co-purified DTaP group versus 9.17% in the DT group. Solicited local adverse events occurred in 21.67% (52/240) versus 7.08% (17/240), including erythema (16.67%), swelling (15.83%), pain (7.08%), induration (6.25%) and pruritus (6.25%). Solicited systemic adverse events occurred in 5.00% (12/240), mainly fever (4.17%); grade ≥3 vaccine-related adverse events occurred in only 0.83% (2/240), and no vaccine-related serious adverse events or deaths were reported (Tang et al., 2026). In a 2026 randomized infant trial of three-component acellular pertussis vaccines, total adverse reactions within 30 days were 16.59%, 16.91% and 14.52% in the DTcP-1, DTaP-IPV-Hib and DTcP-2 groups, respectively; grade ≥3 reactions were 0.66%, 0.66% and 0.59%, respectively. Approximately 90% of reactions resolved within seven days, and no vaccine-related serious adverse events were reported (Zhang et al., 2026).

In adolescents and adults, local pain, erythema and swelling are generally more frequent than after infant primary vaccination, whereas serious events remain uncommon. In German adolescents receiving a sixth consecutive dose of an acellular-pertussis-containing vaccine, Tdap was associated with pain, erythema and swelling in 63.6%, 51.7% and 41.4% of participants, respectively; fever occurred in 9.1%, fatigue in 28.5%, headache in 29.2% and gastrointestinal symptoms in 14.1%. Extensive injection-site swelling occurred in 0.94% (3/319), all resolving spontaneously; one serious adverse event was judged unrelated to vaccination (Zepp et al., 2006). In the SIIPL Tdap trial, solicited local adverse events within days 0-6 occurred in 65.5% of participants, including pain (64.2%), swelling (13.8%) and erythema (10.8%); systemic solicited events occurred in 46.8%, including fatigue (35.8%), headache (25.4%), malaise (14.7%) and dizziness (10.7%). Grade 3 events occurred in 2.1%, and no vaccine-related serious adverse events or deaths were reported (Aydin et al., 2023). In adults with obstructive airway disease, an individual participant data analysis showed injection-site pain in 47.7% and fatigue in 19.3% within four days after Tdap; grade 3 pain and fatigue occurred in 0.9% and 2.8%, respectively, with no new safety signal within one month (Van den Steen et al., 2023).

The safety evidence for maternal immunization supports an acceptable risk-benefit balance, but short-term reactogenicity should be distinguished from pregnancy-outcome signals. In a randomized trial in the United States, no Tdap-related serious adverse events occurred; injection-site reactions were reported in 78.8% of women vaccinated during pregnancy and 80.0% vaccinated postpartum, and systemic symptoms in 36.4% and 73.3%, respectively; infant growth and development were similar (Munoz et al., 2014). In a randomized trial in pregnant Mexican women, mild local pain occurred in 22.2% at 24 hours, local erythema in 2.2% and fatigue in 2.4%; by one month, no adverse reactions were reported, and differences versus controls were not statistically significant (Villarreal Pérez et al., 2017). A systematic review of pertussis vaccination in pregnancy found an increased risk of chorioamnionitis in a pooled analysis of six observational studies (RR = 1.27, 95% CI: 1.14-1.42), but no significant differences in non-pertussis infections, spontaneous abortion, stillbirth or maternal and neonatal death (Andersen et al., 2022). This observational signal does not establish a causal relationship; residual confounding and differences in diagnosis or outcome ascertainment may have contributed to the finding.

Early safety data for next-generation vaccines are also encouraging. In a phase IIa study, a single intranasal dose of the live attenuated BPZE1 vaccine was well tolerated in healthy adults aged 18-49 years, with no serious adverse events, deaths or withdrawals due to adverse events and no persistent nasal shedding at day 29 (Gbesemete et al., 2025). In a phase I trial of the Viaskin-PT epicutaneous patch, PTgen-containing patches were generally safe and no vaccine-related serious adverse events were reported. Local reactions included erythema, pruritus, dryness and mild pain, while systemic reactions included headache, fatigue, abdominal pain, myalgia and subjective fever; most were transient (Chatzis et al., 2021).

Overall, the safety evidence supports three conclusions. First, acellular pertussis vaccines are generally less reactogenic than whole-cell pertussis vaccines, supporting their use in childhood immunization programs. Second, local reactions—particularly pain, erythema, and swelling—are common after booster vaccination and have been reported in approximately 40–65% or more of adolescent and adult recipients. They are generally mild to moderate and short-lived, although reported rates vary across products and studies. Third, serious adverse events, including those considered vaccine-related, and deaths have been uncommon in randomized trials. However, adverse events recorded during follow-up should be distinguished from adverse reactions assessed as related to vaccination, and differences in observation periods and reporting methods limit direct comparisons across studies. The absence of vaccine-related serious adverse events in trials of limited size does not rule out rare risks. Continued post-marketing surveillance is therefore important, particularly for pregnant women, recipients of combination vaccines, coadministration settings, next-generation vaccines, and people with underlying conditions.

## 8 Limitations of the current evidence

Evidence on the immunogenicity, effectiveness, and safety of acellular pertussis vaccines is heterogeneous. Studies differ in vaccine formulation and antigen dose, vaccination schedules and dosing intervals, coadministration with other vaccines, participant age and geographic setting, and duration of follow-up. Laboratory methods also vary, including the assays used to measure antigen-specific antibody responses and, where assessed, functional antibody activity. Because no universally accepted serological correlate of protection against pertussis has been established, differences in antibody concentrations or response rates should not be interpreted directly as differences in clinical protection. Accordingly, immunogenicity findings from studies with different products, schedules, assays, or endpoints are not directly comparable, and comparisons should be interpreted in the context of each study’s design and population.

Safety outcomes are subject to additional methodological variation. Studies may use diaries, telephone contacts, or scheduled visits to collect adverse events; observation periods, reporting after individual doses versus cumulatively, event definitions, severity-grading criteria, and instructions given to parents or caregivers may also differ. These differences can affect the frequency and type of events reported. Although pre-licensure trials are important for assessing common adverse events, they are generally not large enough to reliably detect or compare rare events, including hypotonic-hyporesponsive episodes, seizures, or persistent crying lasting more than 3 hours. Post-marketing surveillance and large observational studies are therefore important for identifying and evaluating potential rare safety signals. Such signals should be interpreted in light of study design and the possibility of confounding; the absence of a signal in a pre-licensure trial does not exclude a rare risk.

Evidence for recently developed or approved pertussis vaccines is more limited and product-specific, particularly with respect to long-term clinical protection and persistence of immune responses. In the absence of an accepted correlate of protection, immunogenicity comparisons with established vaccines can inform evaluation but cannot, by themselves, establish equivalent clinical efficacy. Clinical efficacy trials for new formulations are also increasingly difficult to conduct, and evidence on infection, colonization, and transmission remains limited. Thus, early immunological or clinical endpoints should be interpreted cautiously, and findings from one candidate vaccine should not be generalized to other products.

## 9 Conclusions and perspectives

Acellular pertussis vaccines have important value in reducing reactogenicity, improving acceptability and supporting immunization strategies across age groups. Current evidence supports the use of DTaP, Tdap and related combination vaccines in infants, children, adolescents, adults and pregnant women. Multicomponent vaccines broaden antigenic coverage, maternal immunization provides early passive protection to young infants, and booster vaccination restores waning antibody responses.

Nevertheless, acellular vaccines face persistent challenges, including limited durability, incomplete interruption of transmission, uncertain correlates of protection and antigenic variation. Future research should move beyond antibody concentrations alone and evaluate clinical protection, immune mechanisms and transmission-blocking potential. More robust correlates of protection are needed, including anti-PT IgG, PT-neutralizing antibodies, anti-FHA/PRN/FIM antibodies, functional antibodies, memory B cells, T-cell responses and mucosal immunity.

Real-world effectiveness studies and long-term follow-up should be strengthened. Randomized trials provide high-quality evidence but often have limited exposure opportunities, sample size and follow-up duration. Studies linking immunization records, case surveillance and laboratory confirmation can help evaluate the effectiveness of specific products, schedules and booster strategies in practice.

Antigenic variation should be integrated into future vaccine evaluation. Molecular epidemiology should be used to assess the effects of PRN-deficient strains, PT promoter variants and other antigenic changes on protection by different acellular vaccines and to guide rational antigen selection. Next-generation vaccine development should prioritize mucosal immunity and reduction of transmission. BPZE1 and novel adjuvanted or alternative-delivery vaccines may help address the limitations of conventional intramuscular acellular vaccines.

Maternal and infant schedules also require optimization. Future studies should define the best gestational timing, compare antibody transfer across Tdap products, clarify the clinical relevance of blunting and determine how infant schedules can be adapted to maximize both early protection and durable active immunity. Finally, coverage remains crucial. Physician recommendation, individualized risk communication, targeted strategies for vaccine-hesitant groups and electronic health-record reminders may be more effective than general reminders alone. Pertussis control will require both improved vaccines and stronger implementation of immunization programs.

## References

[ref1] Abu-Raya B. Maertens K. Munoz F. M. Zimmermann P. Curtis N. Halperin S. A. . (2021). Factors affecting antibody responses to immunizations in infants born to women immunized against pertussis in pregnancy and unimmunized women: individual-participant data Meta-analysis. Vaccine 39, 6545–6552. doi: 10.1016/j.vaccine.2021.09.022, 34598822

[ref2] Amirthalingam G. Andrews N. Campbell H. Ribeiro S. Kara E. Donegan K. . (2014). Effectiveness of maternal pertussis vaccination in England: an observational study. Lancet (London, England) 384, 1521–1528. doi: 10.1016/S0140-6736(14)60686-3, 25037990

[ref3] Andersen A. R. Kolmos S. K. Flanagan K. L. Benn C. S. (2022). Systematic review and meta-analysis of the effect of pertussis vaccine in pregnancy on the risk of chorioamnionitis, non-pertussis infectious diseases and other adverse pregnancy outcomes. Vaccine 40, 1572–1582. doi: 10.1016/j.vaccine.2021.02.018, 33642162

[ref4] Aronsson B. Appelqvist E. Jämtberg K. Källberg H. Olin P. Storsaeter J. (2022). Long-term follow-up of the effectiveness of one whole-cell and two acellular pertussis vaccines based on a randomised controlled vaccine trial in Sweden. Acta paediatrica (Oslo, Norway: 1992) 111, 1052–1053. doi: 10.1111/apa.16184, 34773298

[ref5] Aydin I. May M. Pisano F. Mpofu-Maetzig N. Grode L. Parekh S. . (2023). Safety and immunogenicity of SIIPL Tdap, a new tetanus toxoid, reduced diphtheria toxoid and acellular pertussis vaccine, in healthy subjects 4-65 years of age: a phase II/III randomized, observer-blinded, active controlled, multicenter clinical study in Germany. Vaccine 41, 6810–6819. doi: 10.1016/j.vaccine.2023.09.060, 37827966

[ref6] Barug D. Pronk I. van Houten M. A. Versteegh F. G. A. Knol M. J. van de Kassteele J. . (2019). Maternal pertussis vaccination and its effects on the immune response of infants aged up to 12 months in the Netherlands: an open-label, parallel, randomised controlled trial. Lancet Infect. Dis. 19, 392–401. doi: 10.1016/S1473-3099(18)30717-5, 30938299

[ref7] Brousseau N. Angers-Goulet M. Bastien R. Ye L. Sadarangani M. Halperin S. A. (2024). Vaccination during pregnancy and modulation of IgG response to pertussis vaccines in infants: the impact of different vaccine formulations. Vaccine 42, 2138–2143. doi: 10.1016/j.vaccine.2024.03.015, 38461048

[ref8] Calvert A. Amirthalingam G. Andrews N. Basude S. Coleman M. Cuthbertson H. . (2023). Optimising the timing of whooping cough immunisation in mums (OpTIMUM) through investigating pertussis vaccination in pregnancy: an open-label, equivalence, randomised controlled trial. The Lancet. Microbe 4, e300–e308. doi: 10.1016/S2666-5247(22)00332-9, 37080224

[ref9] Chatzis O. Blanchard-Rohner G. Mondoulet L. Pelletier B. De Gea-Hominal A. Roux M. . (2021). Safety and immunogenicity of the epicutaneous reactivation of pertussis toxin immunity in healthy adults: a phase I, randomized, double-blind, placebo-controlled trial. Clin. Microbiol. Infect. 27, 878–885. doi: 10.1016/j.cmi.2020.08.033, 32896653

[ref10] Daniel O. Srikanth S. Clarke P. Le Doare K. Heath P. T. Jones C. E. . (2025). Pertussis antibody responses in infants born to mothers vaccinated at different time points in pregnancy. Vaccine 62:127481. doi: 10.1016/j.vaccine.2025.127481, 40652683

[ref11] Duda-Madej A. Łabaz J. Topola E. Bazan H. Viscardi S. (2025). Pertussis-a re-emerging threat despite immunization: an analysis of vaccine effectiveness and antibiotic resistance. Int. J. Mol. Sci. 26:607. doi: 10.3390/ijms26199607, 41096873 PMC12525418

[ref12] Gao H. Lau E. H. Y. Cowling B. J. (2022). Waning immunity after receipt of pertussis, diphtheria, tetanus, and polio-related vaccines: a systematic review and Meta-analysis. J. Infect. Dis. 225, 557–566. doi: 10.1093/infdis/jiab480, 34543411

[ref13] Gbesemete D. Ramasamy M. N. Ibrahim M. Hill A. R. Raud L. Ferreira D. M. . (2025). Efficacy, immunogenicity, and safety of the live attenuated nasal pertussis vaccine, BPZE1, in the UK: a randomised, placebo-controlled, phase 2b trial using a controlled human infection model with virulent Bordetella pertussis. The Lancet. Microbe 6:101211. doi: 10.1016/j.lanmic.2025.101211, 41344352

[ref14] Goldsmith J. A. Nguyen A. W. Wilen R. E. Wijagkanalan W. McLellan J. S. Maynard J. A. (2025). Structural basis for neutralizing antibody binding to pertussis toxin. Proc. Natl. Acad. Sci. USA 122:e2419457122. doi: 10.1073/pnas.2419457122, 40172968 PMC12002313

[ref15] Gorringe A. R. Vaughan T. E. (2014). Bordetella pertussis fimbriae (Fim): relevance for vaccines. Expert Rev. Vaccines 13, 1205–1214. doi: 10.1586/14760584.2014.930667, 25102891

[ref16] Grimprel E. von Sonnenburg F. Sänger R. Abitbol V. Wolter J. M. Schuerman L. M. (2005). Combined reduced-antigen-content diphtheria-tetanus-acellular pertussis and polio vaccine (dTpa-IPV) for booster vaccination of adults. Vaccine 23, 3657–3667. doi: 10.1016/j.vaccine.2005.02.013, 15882526

[ref17] Hara M. Okada K. Yamaguchi Y. Uno S. Otsuka Y. Shimanoe C. . (2013). Immunogenicity and safety after booster vaccination of diphtheria, tetanus, and acellular pertussis in young adults: an open randomized controlled trial in Japan. Clinical and Vaccine Immunol 20, 1799–1804. doi: 10.1128/CVI.00490-13, 24108779 PMC3889508

[ref18] Heininger U. Cherry J. D. Stehr K. Schmitt-Grohé S. Uberall M. Laussucq S. . Comparative efficacy of the Lederle/Takeda acellular pertussis component DTP (DTaP) vaccine and Lederle whole-cell component DTP vaccine in German children after household exposure. Pertussis Vaccine Study Group. Pediatrics, 102, 546–553 (1998) doi: 10.1542/peds.102.3.546 9738175

[ref19] Hurtaud A. Coomans C. Vuillemin B. Benamar A. Couraud M. Pham B. . (2023). Impact of a dTcaP booster vaccine awareness campaign initiated by the French national health insurance for adults aged 25 years in 2021. BMC Health Serv. Res. 23:903. doi: 10.1186/s12913-023-09805-w, 37612678 PMC10463893

[ref20] Jones C. E. Calvert A. Southern J. Matheson M. Andrews N. Khalil A. . (2021). A phase IV, multi-Centre, randomized clinical trial comparing two pertussis-containing vaccines in pregnant women in England and vaccine responses in their infants. BMC Med. 19:138. doi: 10.1186/s12916-021-02005-5, 34098951 PMC8186138

[ref21] Klein N. P. Abu-Elyazeed R. Cheuvart B. Janssens W. Mesaros N. (2019). Immunogenicity and safety following primary and booster vaccination with a hexavalent diphtheria, tetanus, acellular pertussis, hepatitis B, inactivated poliovirus and Haemophilus influenzae type b vaccine: a randomized trial in the United States. Hum. Vacc. Immunother. 15, 809–821. doi: 10.1080/21645515.2018.1549449, 30444673 PMC6605854

[ref22] Liu X. Wei C. Huang H. Wan J. Li Y. Wang F. . (2025). Preliminarily evaluation the safety and immunogenicity of tetanus, reduced diphtheria and acellular pertussis (five components) combined vaccine, adsorbed (Tdcp) in participants aged 6 years and above: a blinded and randomised, and controlled phase I clinical trial. Front. Immunol. 16:1616574. doi: 10.3389/fimmu.2025.1616574, 40977696 PMC12446275

[ref23] Ma Q. Li T. Gong S. Li J. Wei Y. Hao Y. (2026). A global systematic literature review of Bordetella pertussis anti-PT IgG seroprevalence 2010 to 2023. J. Infect. 92:106708. doi: 10.1016/j.jinf.2026.106708, 41759747

[ref24] Martinón-Torres F. Halperin S. A. Nolan T. Tapiéro B. Perrett K. P. de la Cueva I. S. . (2021). Impact of maternal diphtheria-tetanus-acellular pertussis vaccination on pertussis booster immune responses in toddlers: follow-up of a randomized trial. Vaccine 39, 1598–1608. doi: 10.1016/j.vaccine.2021.02.001, 33612341

[ref25] McAlister S. M. van den Biggelaar A. H. J. Cooper M. N. Thornton R. Richmond P. Marshall H. S. . (2025). Acellular pertussis vaccine given in the week after birth does not impair antibody responses to later childhood doses. Pediatr. Infect. Dis. J. 44, 476–483. doi: 10.1097/INF.0000000000004764, 40063739

[ref26] McGirr A. Fisman D. N. (2015). Duration of pertussis immunity after DTaP immunization: a meta-analysis. Pediatrics 135, 331–343. doi: 10.1542/peds.2014-1729, 25560446

[ref27] Muloiwa R. Kagina B. M. Engel M. E. Hussey G. D. (2020). The burden of laboratory-confirmed pertussis in low and middle-income countries since the inception of the expanded programme on immunisation (EPI) in 1974: a systematic review and meta-analysis. BMC Med. 18:233. doi: 10.1186/s12916-020-01699-3, 32854714 PMC7453720

[ref28] Munoz F. M. Bond N. H. Maccato M. Pinell P. Hammill H. A. Swamy G. K. . (2014). Safety and immunogenicity of tetanus diphtheria and acellular pertussis (Tdap) immunization during pregnancy in mothers and infants: a randomized clinical trial. JAMA 311, 1760–1769. doi: 10.1001/jama.2014.3633, 24794369 PMC4333147

[ref29] Nakabembe E. Greenland M. Amaral K. Abu-Raya B. Amone A. Andrews N. . (2025). Safety and immunogenicity of an acellular pertussis vaccine containing genetically detoxified pertussis toxin administered to pregnant women living with and without HIV and their newborns (WoMANPOWER): a randomised controlled trial in Uganda. Lancet Glob. Health 13, e81–e97. doi: 10.1016/S2214-109X(24)00409-1, 39706666 PMC11659843

[ref30] Nakano T. Hasegawa M. Endo M. Matsuda K. Tamai H. (2024). Immunogenicity and safety of adsorbed diphtheria-purified pertussis-tetanus-inactivated polio (Sabin strain)-Haemophilus type b conjugate combined vaccine (DPT-IPV-Hib) in healthy Japanese infants ≥ 2 and < 43 months of age: a phase III, multicenter, active controlled, assessor-blinded, randomized, parallel-group study. Vaccine 42, 3134–3143. doi: 10.1016/j.vaccine.2023.03.077, 38582691

[ref31] Oguti B. Ali A. Andrews N. Barug D. Anh Dang D. Halperin S. A. . (2022). The half-life of maternal transplacental antibodies against diphtheria, tetanus, and pertussis in infants: an individual participant data meta-analysis. Vaccine 40, 450–458. doi: 10.1016/j.vaccine.2021.12.007, 34949496 PMC9664482

[ref32] Olin P. Rasmussen F. Gustafsson L. Hallander H. O. Heijbel H. (1997). Randomised controlled trial of two-component, three-component, and five-component acellular pertussis vaccines compared with whole-cell pertussis vaccine. Ad hoc Group for the Study of pertussis vaccines. Lancet (London, England) 350, 1569–1577. doi: 10.1016/s0140-6736(97)06508-2, 9393335

[ref33] Pérez Chacón G. Estcourt M. J. Totterdell J. Marsh J. A. Perrett K. P. Campbell D. E. . (2024). Immunogenicity, reactogenicity, and IgE-mediated immune responses of a mixed whole-cell and acellular pertussis vaccine schedule in Australian infants: a randomised, double-blind, noninferiority trial. PLoS Med. 21:e1004414. doi: 10.1371/journal.pmed.1004414, 38857311 PMC11198910

[ref34] Peterson J. T. Zareba A. M. Fitz-Patrick D. Essink B. J. Scott D. A. Swanson K. A. . (2022). Safety and immunogenicity of a respiratory syncytial virus prefusion F vaccine when Coadministered with a tetanus, diphtheria, and acellular pertussis vaccine. J. Infect. Dis. 225, 2077–2086. doi: 10.1093/infdis/jiab505, 34637519 PMC9200146

[ref35] Puthanakit T. Tangsathapornpong A. Anugulruengkitt S. Nantanee R. Bunjoungmanee P. Mansouri S. . (2024). A reduced-dose recombinant pertussis vaccine booster in Thai adolescents: a phase 2/3, observer-blinded, randomised controlled, non-inferiority trial. The Lancet. Child & adolescent health 8, 900–909. doi: 10.1016/S2352-4642(24)00173-1, 39572123

[ref36] Richmond P. Nolan T. McGirr A. Napier-Flood F. Kim J. Leah A. . (2024). Phase 1 trial of an investigational Tdap booster vaccine with CpG 1018 adjuvant compared with Boostrix in healthy adults and adolescents. Vaccine 42:126251. doi: 10.1016/j.vaccine.2024.126251, 39226786

[ref37] Salmaso S. Mastrantonio P. Tozzi A. E. Stefanelli P. Anemona A. Ciofi degli Atti M. L. . (2001). Sustained efficacy during the first 6 years of life of 3-component acellular pertussis vaccines administered in infancy: the Italian experience. Pediatrics 108, e81–e81. doi: 10.1542/peds.108.5.e81, 11694665

[ref38] Sapuan S. Andrews N. Hallis B. Hole L. Jones C. E. Matheson M. . (2022). An observational, cohort, multi-Centre, open label phase IV extension study comparing preschool DTAP-IPV booster vaccine responses in children whose mothers were randomised to one of two pertussis-containing vaccines or received no pertussis-containing vaccine in pregnancy in England. Vaccine 40, 7050–7056. doi: 10.1016/j.vaccine.2022.10.005, 36272877

[ref39] Saso A. Fröberg J. Jobe H. Eleveld M. Okoye M. Kanteh E. . (2026). Mucosal immune responses to Bordetella pertussis in Gambian infants after maternal and primary vaccination: an immunological substudy of a single-Centre, randomised, controlled, double-blind, phase 4 trial. Lancet Microbe 7:101219. doi: 10.1016/j.lanmic.2025.101219 41519134

[ref40] Sereeyothin C. Kerr S. J. Poovorawan Y. Maertens K. Wanlapakorn N. (2025). The effect of preexisting antibodies from Tdap vaccination during pregnancy on infant antibody responses to the pertussis-containing vaccines. Hum. Vaccin. Immunother. 21:2575638. doi: 10.1080/21645515.2025.2575638, 41122946 PMC12548071

[ref41] Sricharoenchai S. Sirivichayakul C. Chokephaibulkit K. Pitisuttithum P. Dhitavat J. Pitisuthitham A. . (2018). A genetically inactivated two-component acellular pertussis vaccine, alone or combined with tetanus and reduced-dose diphtheria vaccines, in adolescents: a phase 2/3, randomised controlled non-inferiority trial. Lancet Infect. Dis. 18, 58–67. doi: 10.1016/S1473-3099(17)30612-6, 29066131

[ref42] Storsaeter J. Hallander H. O. Gustafsson L. Olin P. (1998). Levels of anti-pertussis antibodies related to protection after household exposure to *Bordetella pertussis*. Vaccine 16, 1907–1916. doi: 10.1016/S0264-410X(98)00227-8, 9796042

[ref43] Sun X. Xu Y. Tang F. Xiao Y. Wang Z. Wang B. . (2022). Immunogenicity and safety of concomitant administration of the chinese inactivated poliovirus vaccine with the diphtheria-tetanus-acellular pertussis (DTaP) vaccine in children: a multicenter, randomized, non-inferiority, controlled trial. Front. Immunol. 13:905634. doi: 10.3389/fimmu.2022.905634, 35958596 PMC9361845

[ref44] Tang X. Xiao Y. Chen J. Su Y. Zhou Y. Luo L. . (2026). Immunogenicity and safety of co-purified diphtheria, tetanus and acellular pertussis vaccine in 6-year-old Chinese children. Nat. Commun. 17:214. doi: 10.1038/s41467-025-66890-7, 41318685 PMC12780235

[ref45] Van Damme P. McIntyre P. Grimprel E. Kuriyakose S. Jacquet J. Hardt K. . (2011). Immunogenicity of the reduced-antigen-content dTpa vaccine (Boostrix(®)) in adults 55 years of age and over: a sub-analysis of four trials. Vaccine 29, 5932–5939. doi: 10.1016/j.vaccine.2011.06.049, 21718738

[ref46] Van den Steen P. Cheuvart B. Deraedt Q. Valdes Verelst L. Shamarina D. (2023). Immunogenicity and safety of reduced-antigen tetanus, diphtheria and acellular pertussis vaccination in adults treated for obstructive airway diseases. Hum. Vaccin. Immunother. 19:2159731. doi: 10.1080/21645515.2022.2159731, 36746754 PMC9980557

[ref47] Villarreal Pérez J. Z. Ramírez Aranda J. M. Cavazos M. L. O. Osuna M. D. J. Z. Dávila J. P. Elizondo M. R. B. . (2017). Randomized clinical trial of the safety and immunogenicity of the Tdap vaccine in pregnant Mexican women. Hum. Vacc. Immunother. 13, 128–135. doi: 10.1080/21645515.2016.1232786, 27686182 PMC5287315

[ref48] Weston W. M. Friedland L. R. Wu X. Howe B. (2012). Vaccination of adults 65 years of age and older with tetanus toxoid, reduced diphtheria toxoid and acellular pertussis vaccine (Boostrix(®)): results of two randomized trials. Vaccine 30, 1721–1728. doi: 10.1016/j.vaccine.2011.12.055, 22212127

[ref49] Wilkinson K. Righolt C. H. Elliott L. J. Fanella S. Mahmud S. M. (2021). Pertussis vaccine effectiveness and duration of protection—a systematic review and meta-analysis. Vaccine 39, 3120–3130. doi: 10.1016/j.vaccine.2021.04.032, 33934917

[ref50] Xu Y. Chen H. Wang B. Zhu X. Luo L. Wang S. . (2023). Immunogenicity and safety of concomitant administration of the Sabin-strain-based inactivated poliovirus vaccine, the diphtheria-tetanus-acellular pertussis vaccine, and measles-mumps-rubella vaccine to healthy infants aged 18 months in China. Int. J. infectious Dis 137, 9–15. doi: 10.1016/j.ijid.2023.10.006, 37832931

[ref51] Yu G. Yang W. Ma Y. Zhang N. Tang D. Jin Y. . (2025). Innovative adjuvant strategies for next-generation pertussis vaccines. Hum. Vaccin. Immunother. 21:2545636. doi: 10.1080/21645515.2025.2545636, 40801273 PMC12351750

[ref52] Zepp F. Knuf M. Habermehl P. Mannhardt-Laakmann W. Howe B. Friedland L. R. (2006). Safety of reduced-antigen-content tetanus-diphtheria-acellular pertussis vaccine in adolescents as a sixth consecutive dose of acellular pertussis-containing vaccine. J. Pediatr. 149, 603–610.e1. doi: 10.1016/j.jpeds.2006.06.016, 17095328

[ref53] Zhang L. Prietsch S. O. Axelsson I. Halperin S. A. (2011). Acellular vaccines for preventing whooping cough in children. Cochrane Database Syst. Rev. 1:CD001478. doi: 10.1002/14651858.CD001478.pub4, 21249646

[ref54] Zhang W. Wei C. Wan P. Feng G. Wang F. Wang L. . (2026). Safety and immunogenicity of diphtheria, tetanus and pertussis (acellular, three components) combined vaccine, adsorbed after three-dose priming in 2 months age infants: a randomized, blinded, controlled phase III clinical trial in China. Emerging microbes infections 15:2625556. doi: 10.1080/22221751.2026.2625556, 41615771 PMC12912230

[ref55] Zhu Y. Zhang W. Hu J. Luo S. Zhou Y. Tang X. . (2024). Seroprevalence of IgG antibodies against pertussis toxin in the Chinese population: a systematic review and meta-analysis. Hum. Vaccin. Immunother. 20:2341454. doi: 10.1080/21645515.2024.2341454, 38695296 PMC11067989

